# Design of Novel Nanoemulsion Formulations for Topical Ocular Delivery of Itraconazole: Development, Characterization and *In Vitro* Bioassay

**DOI:** 10.34172/apb.2022.009

**Published:** 2021-02-06

**Authors:** Saba Mehrandish, Shahla Mirzaeei

**Affiliations:** ^1^Student Research Committee, Kermanshah University of Medical Sciences, Kermanshah, Iran.; ^2^Nano Drug Delivery Research Center, Health Technology Institute, Kermanshah University of Medical Sciences, Kermanshah, Iran.; ^3^Pharmaceutical Sciences Research Center, Health Institute, Kermanshah University of Medical Sciences, Kermanshah, Iran.

**Keywords:** Antifungal drugs, Bioassay, Itraconazole, Nanoemulsion, Ocular drug delivery

## Abstract

***Purpose:*** The objective of this study was to design and develop nanoemulsion formulations of Itraconazole (ITZ), a water-insoluble, potent antifungal drug using the spontaneous emulsification method, to improve the ocular delivery and achieve a sustained release of the drug.

***Methods:*** The oil was selected on the basis of the ITZ solubility while the surfactant and co-surfactant were selected based on the thermodynamic stability and globule size. Following the selection of components, a pseudo-ternary phase diagram was constructed for the most promising formulation (F11) using benzyl benzoate (BB) as the oil, Eumulgin CO40 as the surfactant, and propylene glycol as the co-surfactant, by the design of experiments (DoE).

***Results:*** F7 and F11 formulations were found to have an average globule size of 223.5 ± 10.7 nm and 157.5 ± 14.2 nm, besides thermodynamic stability and suitable physicochemical properties. F11 possessed an almost seven-fold higher cumulative percentage of in vitro released ITZ, in comparison to ITZ aqueous suspension after 24 hours. The release data suggested that the best fitted kinetical model for F11 and F7 was the Higuchi and Korsmeyer-Peppas model.

***Conclusion:*** The extended-release of the drug beside an acceptable amount of loaded ITZ suggested that nanoemulsion is suitable for the delivery of the ITZ.

## Introduction

Topical drug delivery is the most popular route of drug administration, because of advantages such as high patient compliance, non-invasiveness, painlessness, and reduced side effects in comparison to systemic forms.^1^ However, the main challenge in the case of topical drug delivery is the low amount of the permeated drug to the target site and achieving the desirable concentration at that site. Due to the limitations of topical ocular forms, treatment with these forms usually requires frequent administration of the drug for a long duration of time to achieve a therapeutic concentration in eye tissues. This long duration is not usually acceptable by patients and can also lead to unexpected side effects.^2^ Novel drug delivery systems have the ability to enhance the pharmacokinetics and intraocular bioavailability of the drug by increasing the surface to volume ratio of the drug or the residence time of the drug on the surface of the eye.^1,3^ Recently, a variety of Nano drug delivery systems like nanoparticles,^4^ nanostructured lipid carriers,^5^ liposomes, nanoemulsions, nanosuspensions, and nanofibers are designed for enhancing drug delivery in different routes of administration.^6-10^ According to the previous studies, nanoemulsions are suitable systems for improving the delivery of lipophilic drugs by making them more water-soluble.^6,11^ They also have advantages like improving the intraocular bioavailability due to a greater surface, fewer side effects because of more targeted delivery, being nontoxic in nature, physical stability, and the possibility of being formulated in various dosage forms.^6^

Recently, the prevalence of fungal diseases is increased due to a significant rise in the number of people with a suppressed immune system, including human immunodeficiency virus (HIV) positive patients, transplant recipients, and cancer patients.^12^ Eye is an organ with a high probability of becoming the target of fungal infections due to its exposure to the external environment and pathogens.^13^ According to the World Health Organization (WHO) report, corneal fungal infection is a major condition that can cause vision loss. As a result, ocular fungal infections should be treated immediately.^14^ Itraconazole (ITZ) is an antifungal agent, with a broad spectrum of antifungal action against different species such as *Aspergillus* species, *Candida* species, and molds. The mechanism behind the ITZ antifungal effect is the inhibition of cytochrome P-450 enzymes leading to the limitation of lanosterol demethylation which is essential for ergosterol synthesis. Inhibition of ergosterol synthesis results in the alteration of fungal membrane permeability triggering the antifungal action. ITZ has the advantage of a much higher affinity to fungal P-450 enzymes compared to ketoconazole. Also, it is reported to have an IC50 of 1.2 μM.^15,16^ There are a few commercial dosage forms of ITZ for ocular drug delivery.^17^ ITZ is a highly hydrophobic drug (water solubility = 0.00964 mg/mL) with a poor intraocular bioavailability when used in a conventional form.^18^

In this study, nanoemulsions were designed and optimized using the design of experiment (DoE) method to develop nanoemulsions containing ITZ with an acceptable drug loading. The selection of optimized formulations containing nontoxic levels of biocompatible surfactant and co-surfactant was the main challenge of this study. The most biocompatible components for ocular application were selected. Also, the concentrations were optimized to cause minimum irritancy to the eye. The prepared nanoemulsions were characterized for physicochemical properties. It was expected that formulating ITZ to nanoemulsion could enhance the intraocular bioavailability of this drug as a result of improved solubility, prolonged residence time, and sustained release of the drug.

## Materials and Methods

### Materials

Itraconazole (Sigma-Aldrich), polyvinyl alcohol (PVA) Mw=72000, (Merck**)**, Tween 80, Tween 20, Span 60 (Merck), Eumulgin CO40 (BASF), Cremophor RH 60 (BASF), Pluronic F127 (BASF), propylene glycol (PG) (Merck), benzyl benzoate (BB) (Merck), methanol (Merck), dichloromethane (DCM) (Merck), ethanol (Merck). Sabouraud 4% Dextrose Agar (SDA) (Merck), Sodium Dihydrogen Phosphate Dodecahydrate (Merck). Deionized distilled water was prepared using an in-house distillation assembly.

### Methods

### Oil solubility study

The solubility of ITZ in different oils was measured by dissolving an excess amount of ITZ in 0.5 mL of different oils. The mixture was kept in a Unimax 1010 DT shaking incubator (Heidolph, Schwabach, Germany) at 36°C and 100 rpm for 24 hours. The samples were then removed from the shaking incubator and centrifuged at 9000 rpm for 10 minutes in a Hettich Zentrifugen Mikro 120. The supernatant was filtered through a 0.22 µm filter, and then diluted with DCM; the samples were then analyzed by UV–Visible spectrophotometer (Model No. UV-Mini 1240, Shimadzu, China) at λmax of 266 nm to estimate the amount of ITZ dissolved in particular oil. Finally, the oil with the highest amount of dissolved drug was selected for preparing the nanoemulsion.^19^

### Selection of surfactants

Different formulations were prepared using the selected oil, PVA solution (0.2 or 1% w/v) as solubility enhancer in the aqueous phase, and different surfactants including Tween 80, Tween 20, Span 60, Eumulgin CO40, Cremophor RH 60, and Pluronic F127 (1 or 5% v/v). The formulations were observed for any sign of instability until fifteen days and characterized for globule size. The most stable emulsion with a suitable globule size was selected for the preparation of the nanoemulsion (Table 1).

**Table 1:** Different pre-formulation prepared to choose optimal surfactants

**Formulations**	**Surfactants and Co-surfactants**								**Size (nm)**	**PDI**
	**PVA**	**Tween 20**	**Tween 80**	**Span 60**	**Eumulgin CO40**	**Cremophor RH 60**	**Pluronic F127**	**PG**		
F1	0.2%	---	---	---	---	---	---	5%	-	-
F2	1%	---	---	---	---	---	---	5%	-	-
F3	0.2%	1%	---	---	---	---	---	5%	483.2	0.437
F4	1%	1%	---	---	---	---	---	5%	425.5	0.423
F5	1%	5%	---	---	---	---	---	5%	29.28	0.453
F6	0.2%	---	1%	---	---	---	---	5%	336.5	0.521
F7 *	1%	---	1%	---	---	---	---	5%	244.2	0.408
F8	1%	---	5%	---	---	---	---	5%	22.3	0.434
F9	1%	---	---	1%	---	---	---	5%	2543	0.825
F10	1%	---	---	5%	---	---	---	5%	2918	1.000
F11 *	1%	---	---	---	5%	---	---	5%	136.7	0.442
F12	1%	---	---	---	---	5%	---	5%	3654	1.000
F13	1%	---	---	---	---	---	5%	5%	2834	0.978

* Optimized formulations.

### Selection of co-surfactant

The co-surfactant was selected based on the thermodynamic stability of the prepared emulsions. Different emulsions were prepared using PG or ethanol (1-5% v/v) as the co-surfactant, then were observed for fifteen days for any sign of thermodynamic instability. Finally, based on the droplet size and stability of the emulsion, the main co-surfactant was selected.

### Construction of pseudo-ternary phase diagram

Following the selection of surfactant and co-surfactant, the DoE was used to construct a pseudo-ternary phase diagram for the nanoemulsion with best globule size, polydispersity index (PDI), and thermodynamic stability, consisting of BB as oil phase, Eumulgin CO40 and PG as surfactant mixture (Smix 1:1, 1:3, 3:1), and 1% w/v PVA solution as the aqueous phase. DoE is one of the most common methods to evaluate the effects of variables, simultaneously on a specific response which has the advantages of reducing the number of required experiments and predicting all of the possible interactions between variables and their effects on the final response. This method was adopted in various papers.^20^ The design of mixtures is a branch of DoE which is applicable to prepare multicomponent systems like emulsions. In this study, the experiments were designed using Minitab® 18 software. Three variables including the percentage of the oil phase, Smix, and the aqueous phase containing PVA 1% solution as a stabilizer were changed simultaneously in the specific ranges determined by the pre-formulation studies (oil phase: 1 to 5%, Smix: 1 to 30%, aqueous phase: 65 to 99%) and 27 experiments were designed using Extreme vertices design. Finally, these 27 mixtures were prepared in the laboratory randomly and were analyzed for the globule size as the main response. The pseudo-ternary phase diagram was constructed using the obtained responses by ProSim software (Table 2).

**Table 2:** The experiments designed by the Minitab® 18.1 software using the Extreme vertices designing method

**Order of experiments**	**Code of experiment**	**Smix** **(%)**	**Oil** **(%)**	**Aqueous phase (%)**	**Size (nm)**		
					**Smix (1:1)**	**Smix (1:3)**	**Smix (3:1)**
1	1	1	1	98	759.0	348.5	172.6
4	2	1	5	94	133.6	114.6	163.7
7	3	30	1	69	30760	751.0	812.5
6	4	30	5	65	428.5	247.5	166.5
5*	5	15.5	3	81.5	149.7	189.9	788.9
3	6	8.25	2	89.75	842.8	154.5	153.6
2	7	8.25	4	87.75	292.0	171.5	353.0
9	8	22.75	2	75.25	4414	164.9	381.4
8	9	22.75	4	73.25	516.8	348.5	172.6

* Optimized formulations.

### Preparation of nanoemulsions

**Figure 1:**
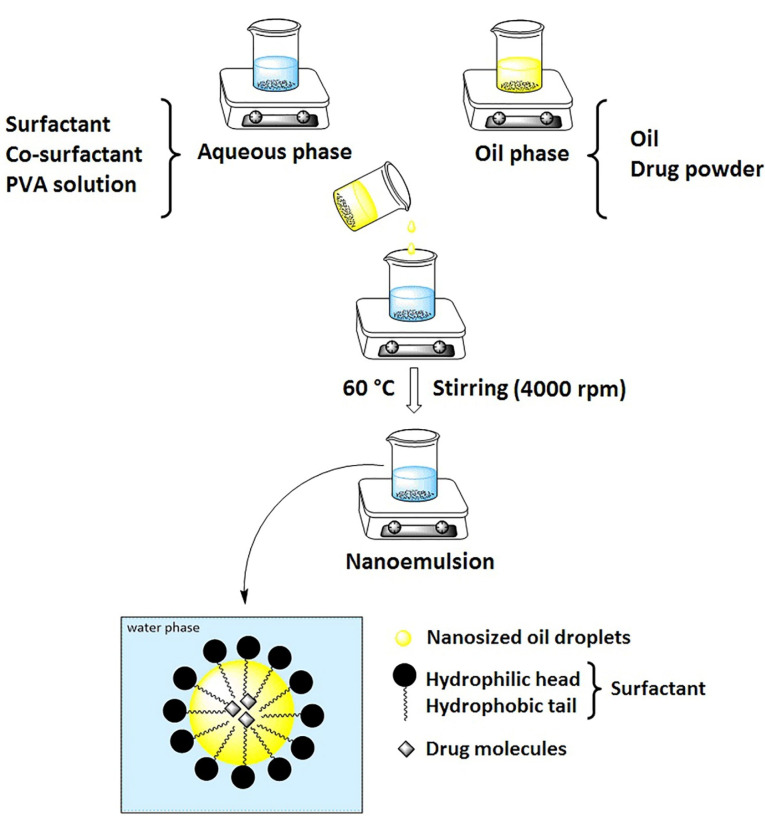
Schematic representation of nanoemulsion preparation using spontaneous emulsification method.

Nanoemulsions were prepared by the spontaneous emulsification method. There is ample research on the preparation of nanoemulsions by this method.^21,22^ The oil and aqueous phases were prepared separately. The oil phase consisted of dissolved ITZ in BB and the aqueous phase contained PVA solution, surfactant, and co-surfactant. To prepare the oily phase, ITZ (5 mg for F11 and 1 mg for F7) was dissolved in BB oil (0.6 mL for F11 and 0.2 mL for F7). The aqueous phase was prepared by dissolving surfactants and co-surfactants (7.5% w/v of Eumulgin CO40 and 7.5% v/v PG in F11 and 1% v/v of tween 80 and 5% v/v PG in F7) in 20 mL of PVA solution (1% w/v) (Table 1). The oil phase was added to the aqueous phase dropwise with continuous stirring (4000 rpm) using a Stirring plate (IKA RCT basic, IKA RH basic 2) at 60°C. It should be noted that ITZ is thermally stable at this temperature.^23^ Finally, we continued to stir the mixture for 60 minutes at 25°C and the nanoemulsion was prepared (Figure 1).

### Globule size and zeta potential analysis

The zeta potential, average globule size, and PDI of the optimized formulations were analyzed at 25 °C using Zeta-sizer ZS Nano (Malvern Instruments, Herrenberg, Germany).

### Stability tests

*Centrifuge:* The optimized nanoemulsions were centrifuged (Rotofix 32, Hettich Zentrifugen, Germany) at 4000 rpm for 30 minutes and then was observed for cracking, creaming, or phase separation. If the prepared emulsion didn’t show any sign of instability, they were subjected to heating-cooling and freeze-thaw cycles.

*Heating-cooling cycle:* six cycles of heating and cooling were done with the storage of samples for at least 48 hours at every temperature and the temperature was changed from 4°C to 45°C.

*Freeze-thaw cycle:* Three Freeze-Thaw cycles with changing the temperature from -20°C to 25°C were done. The samples were stored for at least 48 hours at every temperature.

Finally, the samples were observed for any sign of instability and then analyzed for changes in the globule size.^24,25^

### Microbial assay

Candida albicans ATCC (American Type Culture Collection) 10231 was activated in Sabouraud dextrose broth. The Microbiological assays were performed on Candida albicans by standard disk diffusion method. SDA plates were used to cultivate fungi. The spread-plate method was adopted^26^ and the culture medium was incubated at 28°C for 24 hours. The fungal suspension was applied uniformly on the surface of an SDA plate before the placement of the disks. Standard ITZ solutions with different concentrations (S_1_=1000, S_2_=500, S_3_=250, …, S_13_=0.24 μg/mL) were prepared. Sterile paper disks of 5mm diameter containing 30 μL of standard ITZ solution were placed in plates then incubated at 25°C for 24 hours, after which the average diameter of the inhibition zone surrounding the disks was measured in mm using Vernier caliper. The experiment was repeated twice with three replicates for each concentration. The calibration curve was drawn based on the inhibitory zones obtained from S_3_ to S_13_ and the regression equation of y = 0.3433x + 1.2558 with R^2^ being equal to 0.9876 used for the measurement of the drug in the further studies.

### Drug loading assay

The drug loadings of optimized formulations were measured using the microbial assay method. Sterile disks containing a specific amount of nanoemulsions were placed on SDA plates after the application of fungal suspension. The measured amount of drug was compared to the theoretical amount to estimate the drug loading.

### Viscosity, pH, and conductivity determination

The viscosity, pH, and conductivity of the optimized formulations were determined using a Brookfield DV-III Ultra programmable Rheometer (Brookfield Engineering Laboratories, Inc. II Commerce Blvd, Middleboro, MA, USA), pH meter (827 pH lab, Metrohm, Swiss), and conductivity meter (WTW GmbH, Weilheim, Germany, model: inoLab® Cond 7110). Mean values were reported for each test.

### In vitro drug diffusion study and release kinetics

In order to perform the *in vitro* drug diffusion study, three assemblies were arranged using ITZ aqueous suspension, F7, and F11. A specific amount of each formulation containing the equivalent of 2.5 mg ITZ was filled inside a Falcon tube that was closed at one end with a dialysis membrane (as the acceptor compartment) and was then placed in 40 mL of phosphate buffer (pH 7.4) containing 5% v/v of tween 20 and 30% v/v of isopropanol at 37°C^27^ (as receptor compartment).^19^ At specific time intervals, samples were withdrawn, with full replacement of the receptor medium (to remain the sink condition). The samples were then assayed through the microbial assay method. The release kinetics was modeled by fitting the release data to the zero-order, first-order, Higuchi, Hixon-Crowell, and Korsmeyer-Peppas models and comparing the R^2^ corresponding to each model.

## Results and Discussion

### Oil solubility study

**Figure 2:**
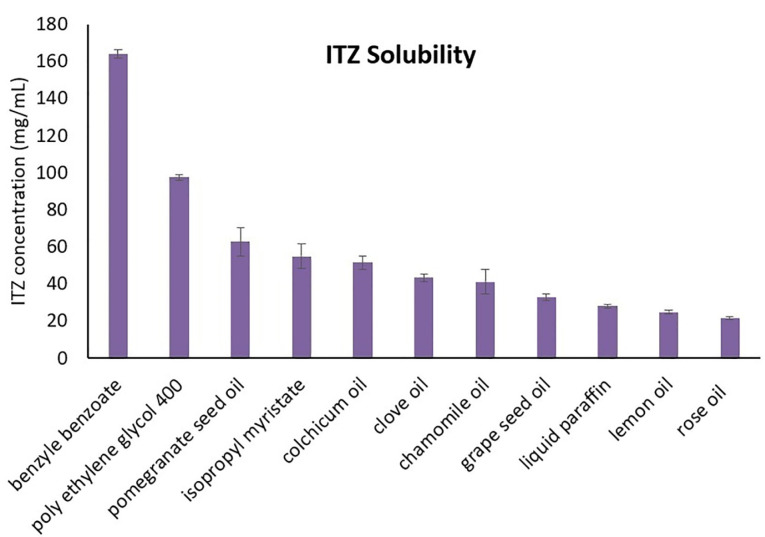
Measured ITZ solubility in different oils after shaking and incubation at 36°C (100 rpm) for 48 hours.

As previously mentioned, ITZ is highly water-insoluble (water solubility of 0.00964 mg/mL).^18^ Therefore, the selection of the oil with high solubility for ITZ can significantly reduce the instability of the nanoemulsion besides improving the drug delivery.^25^ The solubility study was carried out on rose oil, lemon oil, liquid paraffin, grape seed oil, isopropyl myristate, clove oil, pomegranate seed oil, chamomile oil, colchicum oil, and BB. It was observed that the BB oil showed the best solubility for ITZ (164.018 mg/mL) (Figure 2). This oil is a therapeutic agent employed to treat scabies and lice. Sharma et al. used BB as the oily phase for preparing a nanoemulsion for transdermal drug delivery.^28^ It has been claimed that in lower concentrations this oil is just a little irritant to the eye and does not cause any tissue damage.^29,30^ BB was also applied for the development of Verisome^TM^ which is an ocular drug delivery technology.^31^

### Selection of surfactant and co-surfactant

Different formulations were prepared using one of the Tween 80, Tween 20, span 60 or Eumulgin CO40 (1-5% v/v) surfactants, PVA (0.2 and 1% w/v), and PG (5% v/v). PVA at a concentration of 1% w/v showed the best viscosity and stability so PVA 1% w/v solution was selected for the aqueous phase. Emulsions containing Tween 20-PVA, Tween 80-PVA, and Eumulgin CO40-PVA exhibited stability without any phase separation. The emulsions with more than 1% v/v of Tween 20, Tween 80, and Eumulgin CO40 showed additional Nano-sized globules in the size analysis that seems to be the micelles formed by excess surfactant molecules due to the increase of the surfactant concentration above the CMC. Emulsions prepared with Tween 80 (1% v/v), PVA (1% w/v), and PG (5% v/v) (F7 pre-formulation) and Eumulgin CO40 (5% w/v), PVA (1% w/v), and PG (5% v/v) (F11 pre-formulation) showed the best globule size and PDI (almost 200 nm with a PDI less than 0.4) and was selected as the optimized mixture of surfactants** (**Table 1).

PVAs are synthetic polymers used in various medical and pharmaceutical industries. They are popular nontoxic excipients for preparing different dosage forms and also novel drug delivery systems.^32^ PVA was formerly used to prepare both conventional and novel systems for ocular delivery such as that employed by Jain et al. to prepare ocular ciprofloxacin inserts.^33^ Tween 80 or polysorbate 80 is a non-ionic surfactant in the pharmaceutical, food, and cosmetic industries. It has not shown any considerable toxicity except in high doses and can be utilized in pharmaceutical formulations.^20,24^ Earlier studies such as Anjana et al. also used tween 80 as an emulsifier to prepare a nanoemulsion for ocular delivery of curcumin.^34^ In another study, Youshia et al designed a nanoemulsion for ocular delivery of methazolamide via tween 80, which showed good ocular tolerance and was reported to be non-irritant to albino rabbits’ eyes.^35^ Eumulgin CO40 is an emulsifier obtained by the addition of ethylene oxide onto hydrogenated castor oil to stabilize emulsions in different fields.^36^ There was no study on potential damage and toxicity to the ocular tissues by Eumulgin CO40. PG and ethanol (1-5% v/v) were added to the optimized mixture of surfactants as the co-surfactants to ease the preparation of emulsions by lowering the surface tension. It was observed that PG at a concentration of 5% v/v introduced the most stable emulsion. As a result, PG (5% v/v) was selected as the optimal co-surfactant.

PG is also widely applied to prepare conventional and novel ocular delivery systems including the new model aqueous ophthalmic solution of indomethacin prepared by Dimitrova et al.^37^ Due to the described reasons, the final nanoemulsions could have the potential to be nontoxic to the eye and ocular tissues.

### Construction of pseudo-ternary phase diagram

Based on the analyzed responses obtained from the experiments by the Minitab® software, the randomization was performed successfully. The pseudo-ternary phase diagram was constructed based on the globule size of the prepared emulsions by the ProSim software. As illustrated inFigure 3, the nanoemulsion could have been prepared in the green areas. It was observed that a surfactant alone could not reduce the interfacial tension to form the nano-sized globules. A co-surfactant which could lead to an enhanced fluidity and flexibility was needed for the penetration of the oil in the hydrophobic region of surfactants.

**Figure 3:**
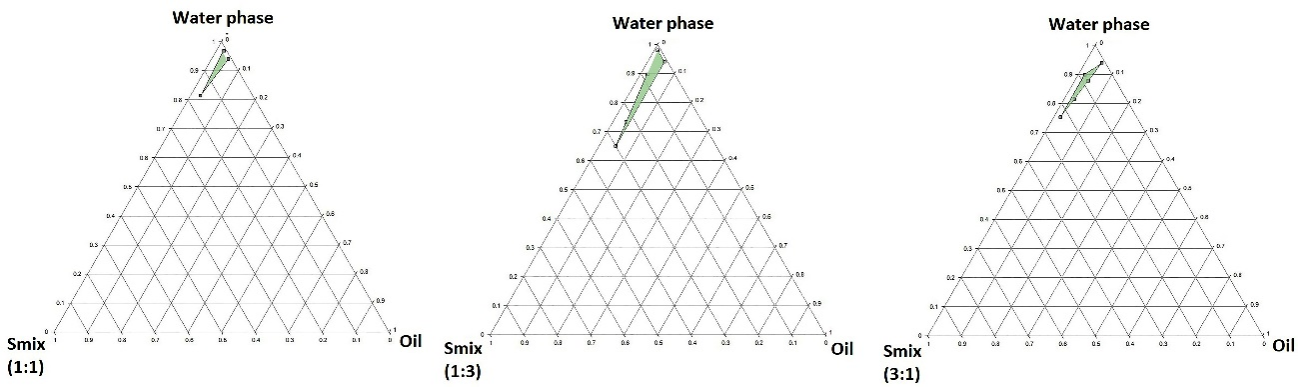
The pseudoternary phase diagram constructed by ProSim software for a mixture of benzyl benzoate as oil phase, Eumulgin CO40 and PG as surfactant and co-surfactant in different Smix ratios (1:1, 1:3, 3:1), and PVA (Mw=72.000) 1% w/v solution in water as the stabilizer.

Increasing the ratio of surfactant to co-surfactant from 1:3 to 3:1 led to the depletion of the nanoemulsion area caused by the reduction of co-surfactant concentration. The Smix ratios above 1:1 led to thicker viscosity reducing fluidity and flexibility of the aqueous phase. Finally, it was observed that the widest nanoemulsion area was achieved by a 1:3 Smix ratio. However, the 1:1 Smix was chosen contained the minimum optimized concentrations of both surfactant and co-surfactant, to prevent irritancy for the eye.

It seems that the optimized nanoemulsion consisted of 15.5% Smix (1:1), 3% oil phase, and 81.5% aqueous phase, which was formed spontaneously and showed thermodynamic stability.^38,39^

### Drug loading assay

Drug loading was expressed as the ITZ amount found in the formulation to the theoretical quantity of the drug. The amount of ITZ measured by microbial assay for a specific amount of F7 was 1.39±0.21 µg while the quantity of ITZ used for preparing formulation was 1.5 µg, so the drug loading percentage for this formulation was 92.84%. The amount of ITZ measured by microbial assay for a specific amount of F11 was 6.98±0.53 µg, while the quantity of ITZ for preparing formulation was 7.5 µg, so the drug loading percentage for this formulation was 93.14%. Both formulations have shown almost accurate drug loading, which confirms the integrity of the formulations.

### Stability tests

**Figure 4:**
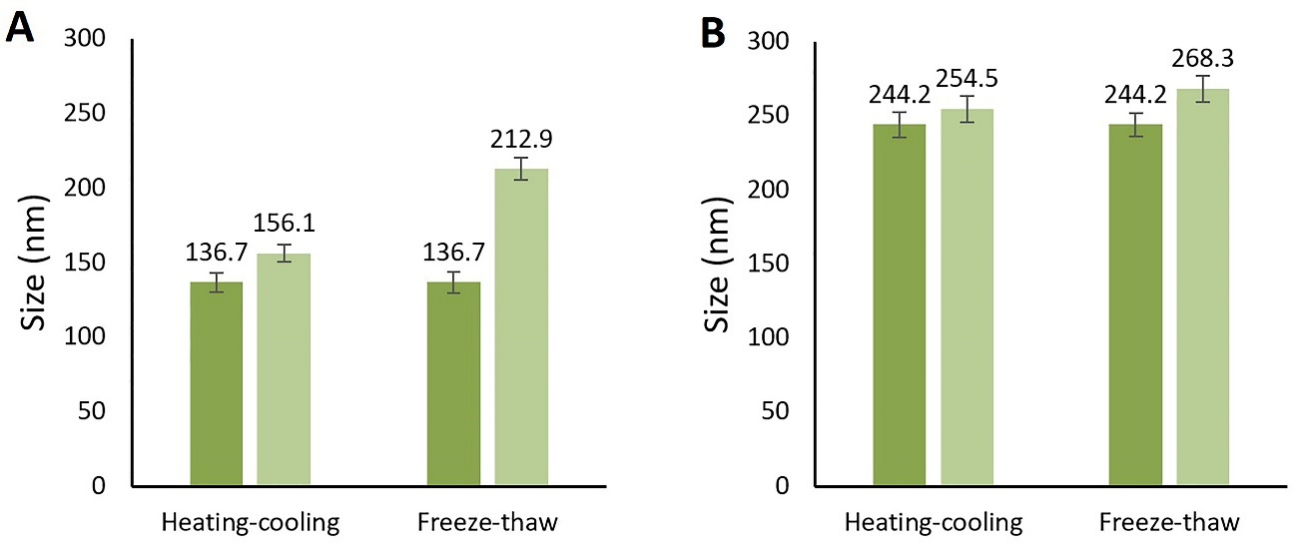
Globule size change after the Heating-cooling and freeze-thaw cycles. A: F11 formulation, B: F7 formulation.

Nanoemulsions are thermodynamically and physically stable systems, made from an optimized mixture of surfactants, oil, and water so they are resistant to any instability such as phase separation, size changing, etc.^40^ In this study, the stability tests including centrifuge test, three cycles of freeze-thaw test, and six cycles of the heating-cooling test were performed on the optimized nanoemulsions. At the end of the tests, there could not be detected any sign of instability and significant size or PDI changing in both F7 and F11 nanoemulsions. The optimized formulations were thermodynamically stable and so they were both chosen for the *in vitro* study (Figure 4).

### Particle size and zeta potential analysis

**Figure 5:**
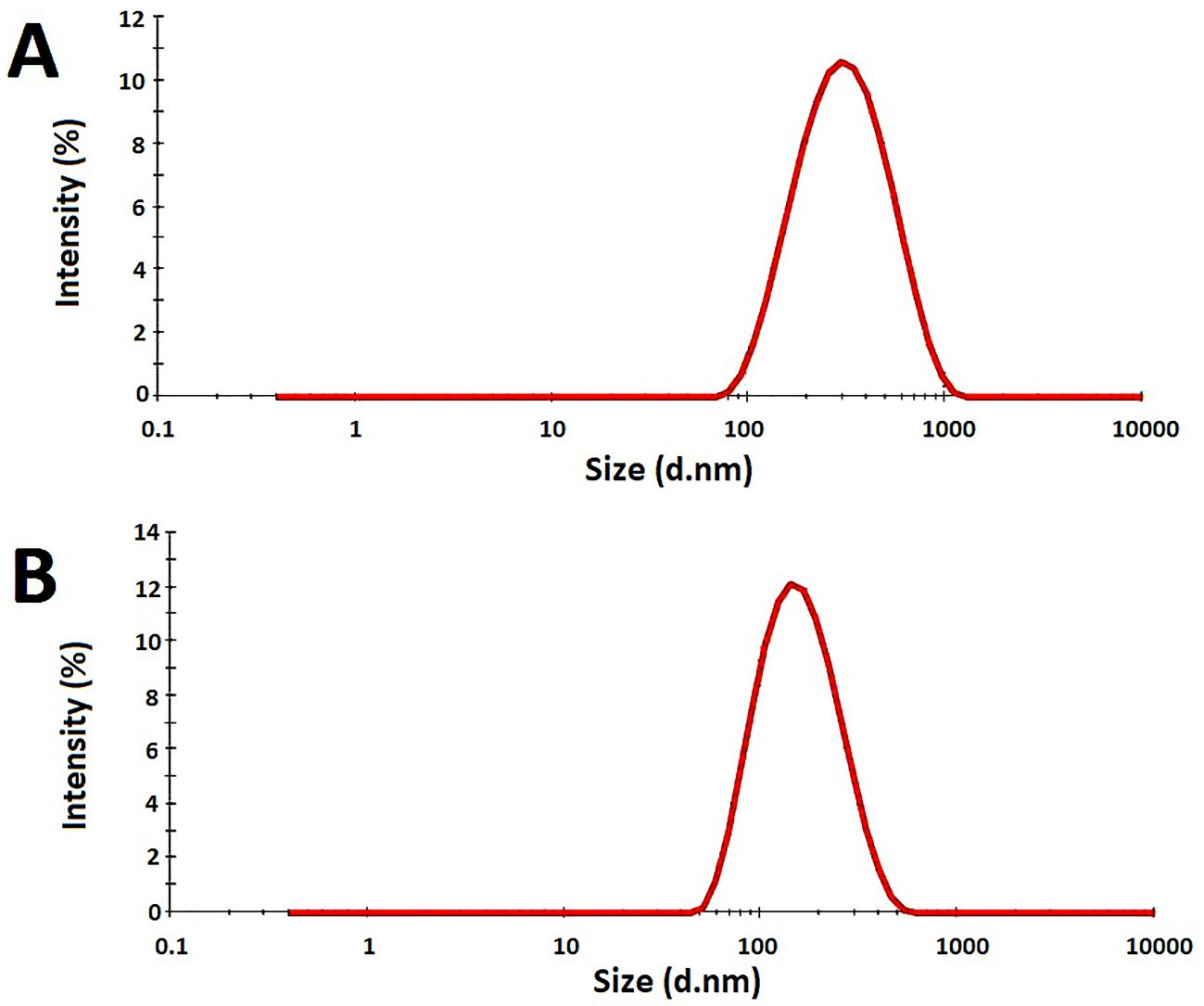
Size distribution of formulations measured by a zeta-sizer at 25°C. A: F7 formulation, B: F11 formulation.

F7 had the mean globule size of223.5 ± 10.7 nm with 100% intensity and PDI of 0.157 ± 0.012 while F11 had the mean globule size of 157.5 ± 14.2 nm with 100% intensity and PDI of 0.233 ± 0.007 (Figure 5). The mean globule sizes were in the range defined for the nanoemulsions and the measured PDIs showed suitable uniformity of the oil droplets. It seems that the Eumulgin CO40 has made a stronger barrier against the coalescence by emulsifier than tween 20. In fact, the Eumulgin CO40 is more powerful in lowering the interfacial tension between the oil and water phases. The mean zeta potential of F7 was -9.2 ± 0.4 while it was -0.623 ± 0.04 for F11. Zeta potential is significantly dependent on the kind of surfactants used for the preparation of the nanoemulsion. Although zeta potential values higher than +20 mV or lower than -20 mV are considered suitable values to ensure good stability of the nanoemulsions by electrostatic repulsion, stable nanoemulsions could be obtained by almost zero zeta potential using nonionic or polymeric surfactants. In this case, stabilization occurred due to steric repulsions.^41^ It seems that the main force in the stabilization of F11 is the steric repulsion while both electrostatic and steric repulsions could be effective in the stabilization of F7. In a previous study, stable nanoemulsions which were emulsified using tween 80 were obtained with a zeta potential of -6.9 ± 0.2 mV by Sari et al.^42^ The negative value of F7 could be due to the presence of tween 80 (Table 3).^43^

**Table 3:** The measured size, zeta potential, conductivity, pH, and viscosity of the optimized nanoemulsion formulations

**Formulations**	**Size (nm)**	**Zeta potential (mV)**	**Conductivity (μS/cm)**	**pH**	**Viscosity (cP)**
F7	223.5 ± 10.7	-9.2 ± 0.4	113.53±0.50	7.12±0.07	2.2±0.1
F11	157.5 ± 14.2	-0.623 ± 0.04	165.46±0.15	5.86±0.1	3.4±0.2

### Viscosity, pH, and conductivity determination

The samples obtained from F7 and F11 had a mean pH of 7.12 ± 0.07 and 6.68 ± 0.1 respectively, which is favorable for ocular application. They also had a mean viscosity of 2.2 ± 0.1 and 3.4 ± 0.2 cP respectively, slightly higher than the viscosity of conventional eye drops which is commonly lower than 2 cP. This enhanced viscosity was enough to increase the corneal residence and contact time of formulations but was not sufficient to resist the distribution on the surface of the eye.^44^ Mean conductivity of 113.53 ± 0.5 and 165.46 ± 0.15 μS/cm was observed for F7 and F11 which has confirmed the existence of O/W nanoemulsions (Table 3).^25,45^

### In vitro drug diffusion study

**Figure 6:**
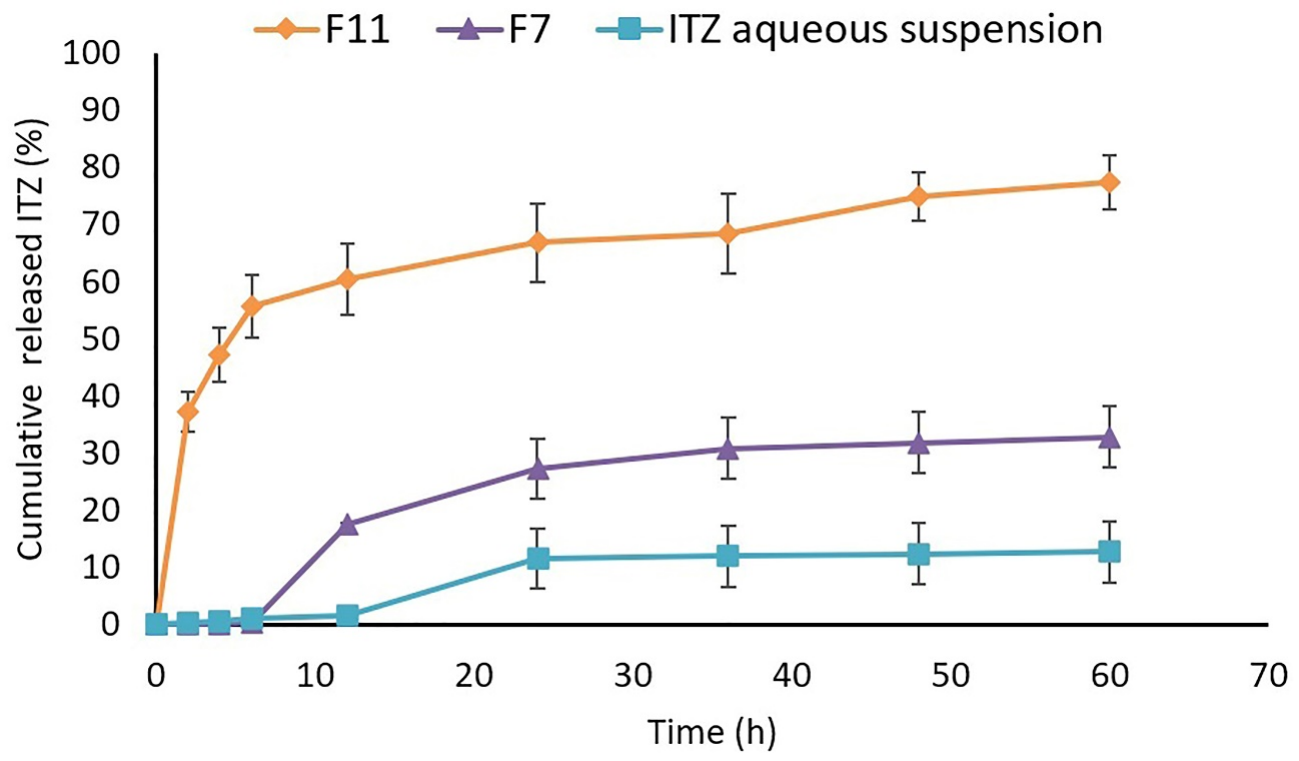
Comparison of *in vitro* drug release profile in nanoemulsions, and aqueous suspension in the first 60 hours of drug release study.

The data obtained from*in vitro* drug diffusion study were shown in Figure 6. It was observed that the total released drug from the optimized nanoemulsions was higher than that of the drug suspension. The F11 released almost 60% of the drug in the first 6 hours while the F7 and aqueous suspension released less than 1% of the drug during the same interval. At the end of the 60 hours, the percentage of the drug released into the receptor compartment for F11, F7, and ITZ aqueous suspension was 77.29 ± 4.685, 32.80 ± 6.247, and 12.71 ± 5.328, respectively. It seems that the reason behind the extended-release of ITZ from nanoemulsions compared to the aqueous suspension was the small size of the globules containing ITZ, which resulted in a larger surface to volume ratio. It also improved drug solubility which could lead to an extended-release of the drug for longer durations.^19^ Despite the almost 2.5 folds greater released drug compared to the aqueous suspension, F7 showed an inappropriate release profile with a long lag time and a low percentage of the released drug. It seems this was because of the lower amount of loaded drug in F7 besides the larger globule size compared to F11. F11 showed a suitable, two-step release profile with an initial burst in the first 6 hours releasing 60% of the drug, followed by a controlled release of the drug. It also released the drug almost 7-folds greater compared to the aqueous suspension (*P* < 0.05).

Finally, by the result obtained from the *in vitro* study, it could be concluded that the optimized nanoemulsion achieves a higher concentration for a prolonged time in comparison to aqueous suspension in ocular delivery which could lead to a less frequent administration of the drug with a reduced dose, which increases patient compliance and reduces the side effects of the drug. Also, the sustained release profile coupled with the enhanced residence time of the formulation on the surface of the eye could enhance the intraocular bioavailability of the poorly soluble drugs.^44^

Similar results were reported by Thakkar et al. who designed a nanoemulsion formulation of ITZ using Capmul MCM C8 as oil, Cremophor EL, and Pluronic F68 as surfactant systems. It was reported that the developed nanoemulsion of ITZ showed a higher percentage of *in vitro* released drug compared to plain drug suspension due to the enhanced surface to volume ratio and solubility.^19^ In another study, nanoemulsions of ITZ were prepared using Eugenol, Sodium cholate, and Lecithin. The results exhibited 40-70% released ITZ in 24 hours. It was claimed that the higher percentage of the released drug in some formulations is due to the smaller mean particle size.^46^

The released data suggested that Higuchi was the best fitted kinetical model for F11 which showed a higher R^2^ value compared to other kinetical models. Nanoemulsions typically followed a zero-order kinetic at the outset which eventually changed to the Higuchi model. To explain, the diffusion of the drug from the nanoemulsion to the receptor medium becomes the rate-limiting step of the drug release after a while.^39,47^ Korsmeyer-Peppas was the kinetical model fitted for F7 which was observed for nanoemulsions in previous studies (Table 4).^25^

**Table 4:** The correlation factors obtained by fitting the release data to the zero-order, first-order, Higuchi, Hixon-Crowell, and Korsmeyer-Peppas models

**Formulations**	**R** ^2^				
	**Zero-order**	**First-order**	**Higuchi**	**Korsmeyer-Peppas**	**Hixon-Crowell**
F7	0.7833	0.9107	0.8845	**0.9346**	0.8763
F11	0.8374	0.8602	**0.9145**	0.8812	0.8527

## Conclusion

Nanoemulsions could be designed to overcome the problems associated with the poor bioavailability of the hydrophobic antifungal drug, ITZ, in ocular delivery. In this study, after the selection of the components with the least ocular toxicity and best globule size and stability, the optimized F7 and F11 formulations were chosen by the pseudo-ternary diagram using the DoE method. The prepared nanoemulsions were thermodynamically stable with a suitable globule size of223.5 ± 10.7 and 157.5 ± 14.2 nm for the F7 and F11, respectively. F11 showed an appropriate release profile by releasing the drug with a percentage of almost 7-fold in 60 hours compared to the aqueous suspension of ITZ. It seems that the globule size reduction and higher surface to volume ratio enhanced the drug release for the nanoemulsion in comparison with the aqueous suspension. Also, the sustained release profile of F11 besides its enhanced residence time on the surface of the eye could lead to an increased intraocular bioavailability in the ocular tissues. It was also previously reported that the more antifungal effect could be associated with an enhanced drug release. Based on the results, it could be concluded that nanoemulsions could be proper systems for ocular delivery of ITZ with increased bioavailability, less frequent administration, and more patient compliance. Still, further studies including the *in vivo* and ophthalmic toxicity studies are required.

## Ethical Issues

The protocol was approved by the Local Ethical Committee of Kermanshah University of Medical Sciences; approval number: IR.KUMS.REC.1396.656.

## Conflict of Interest

The authors declared no conflict of interest in this study.

## Acknowledgments

The authors would like to thank the Research Council of Kermanshah University of Medical Sciences (Grant Number: 96671) for financial support of this work. Also, faithfully thanks Shiva Taghe for her assistance and Rahesh Daru Novin Co. for kind cooperation in providing materials and equipment.
